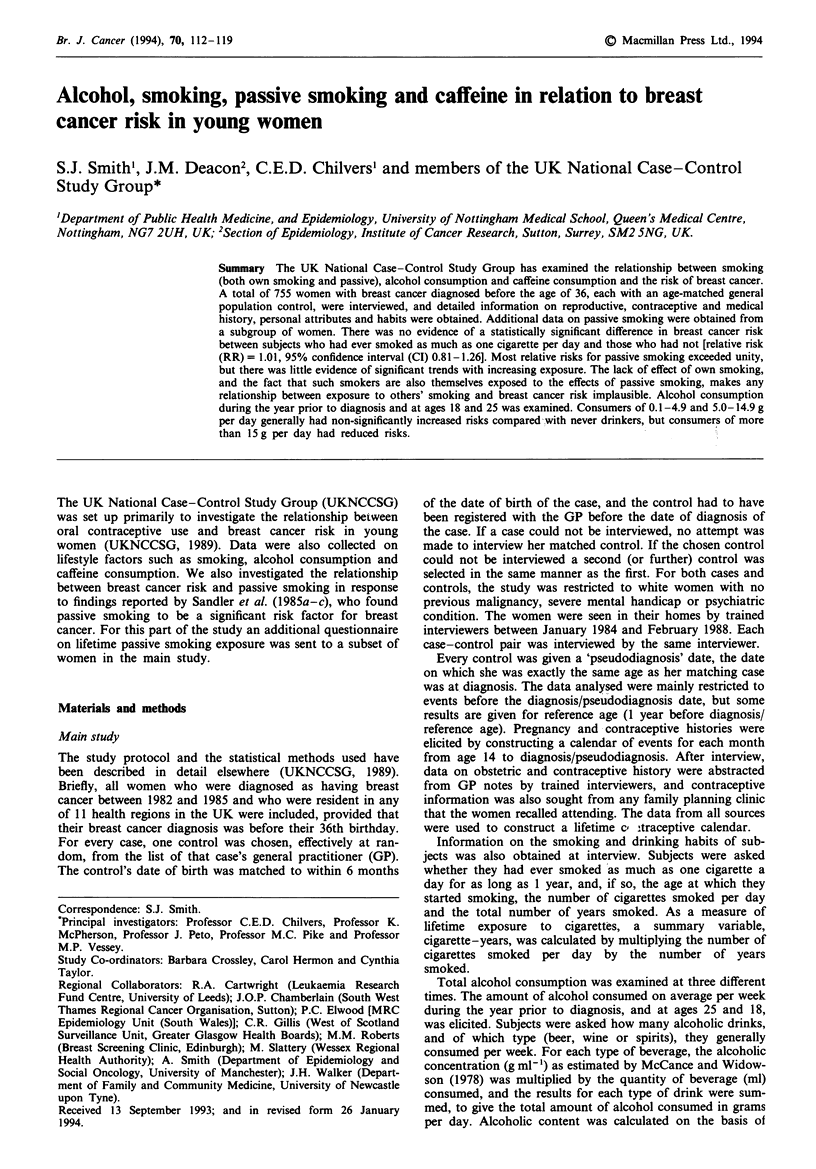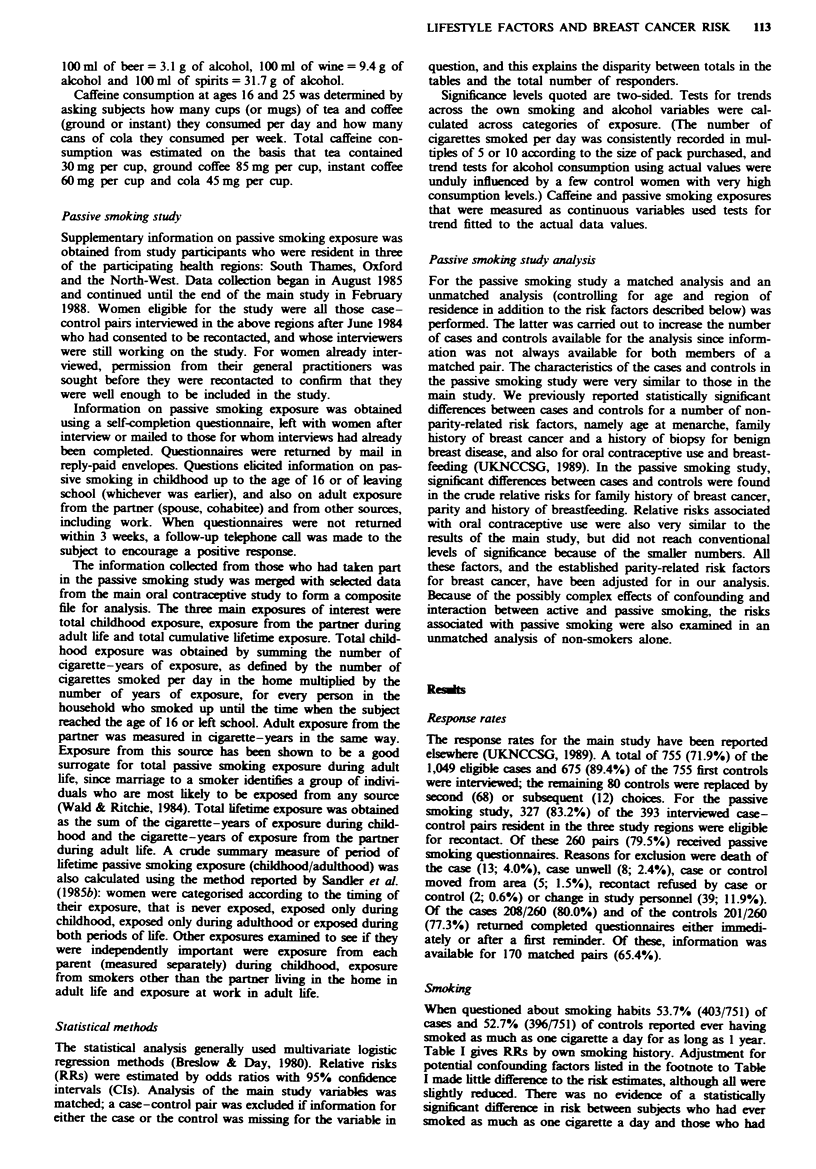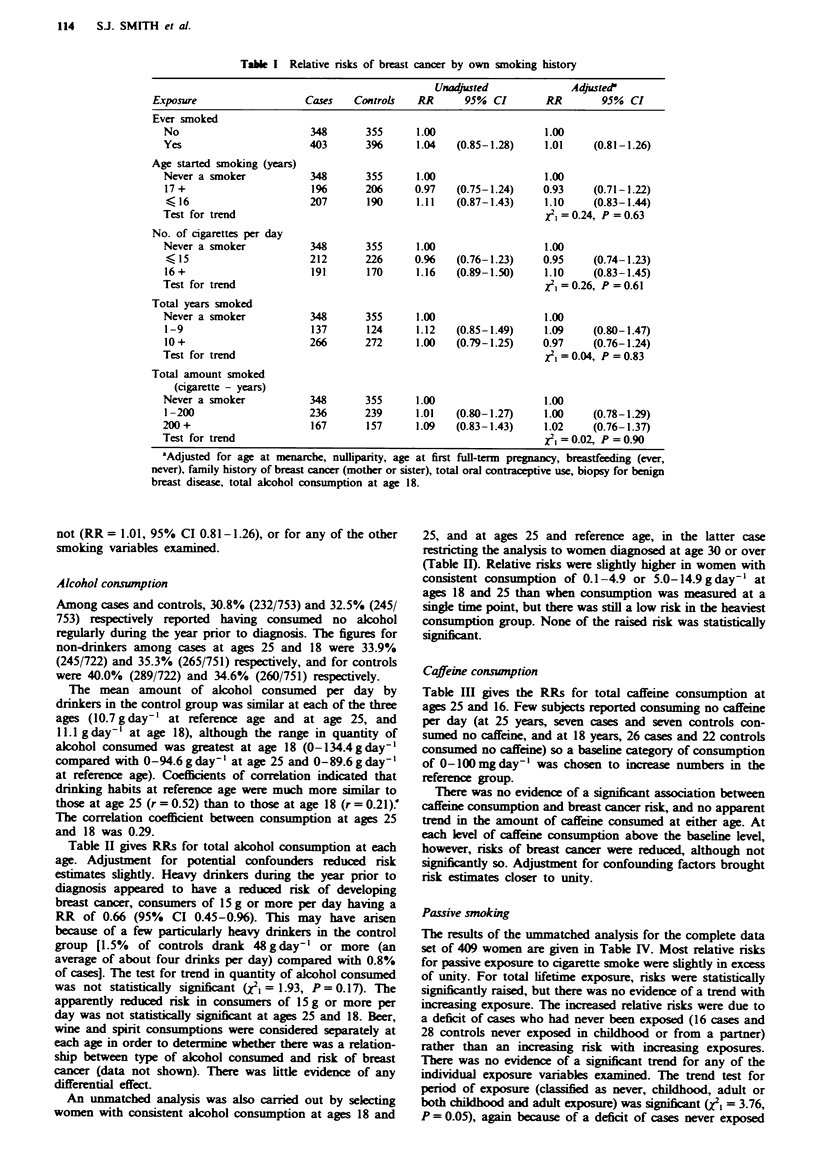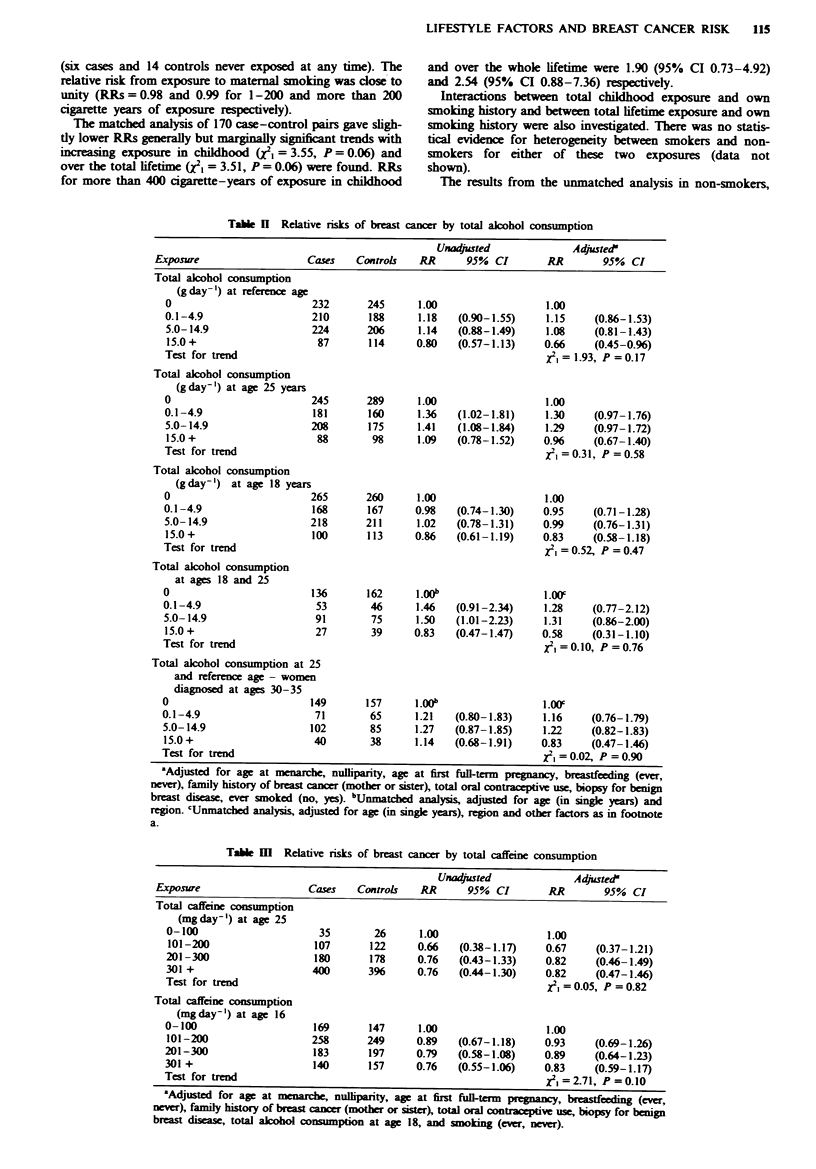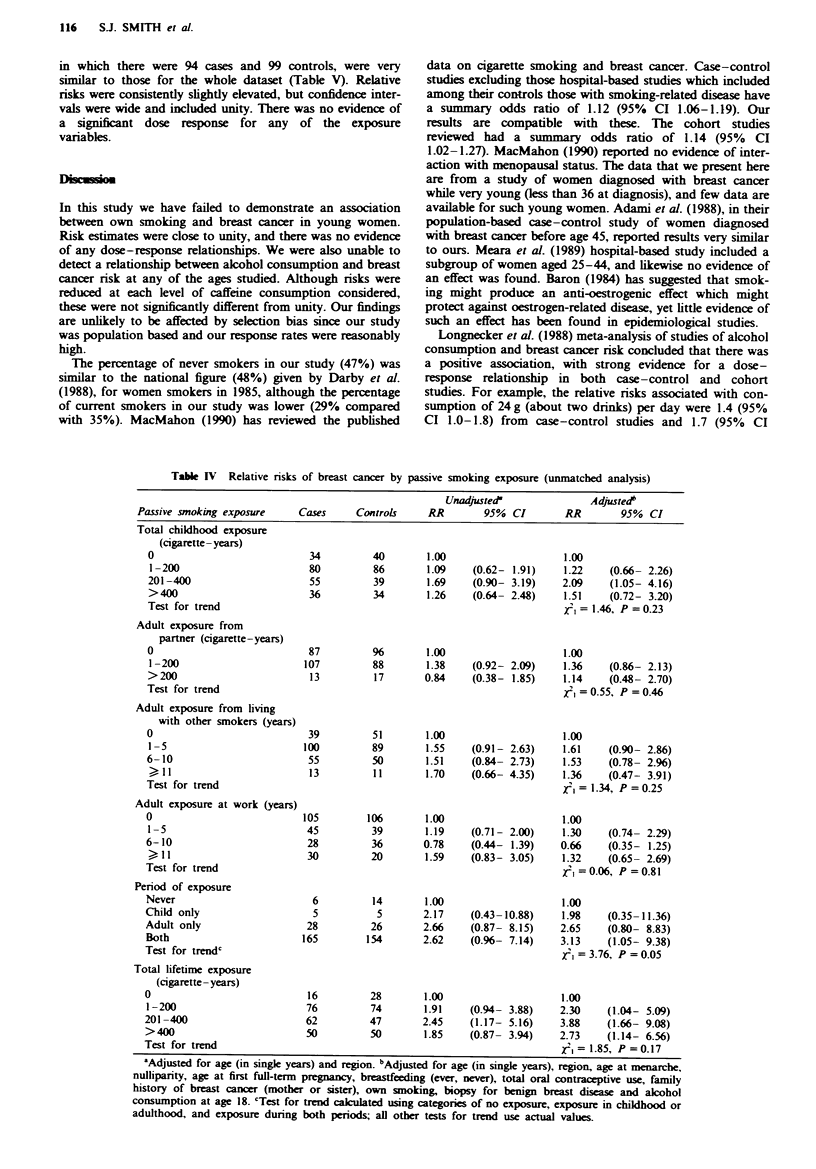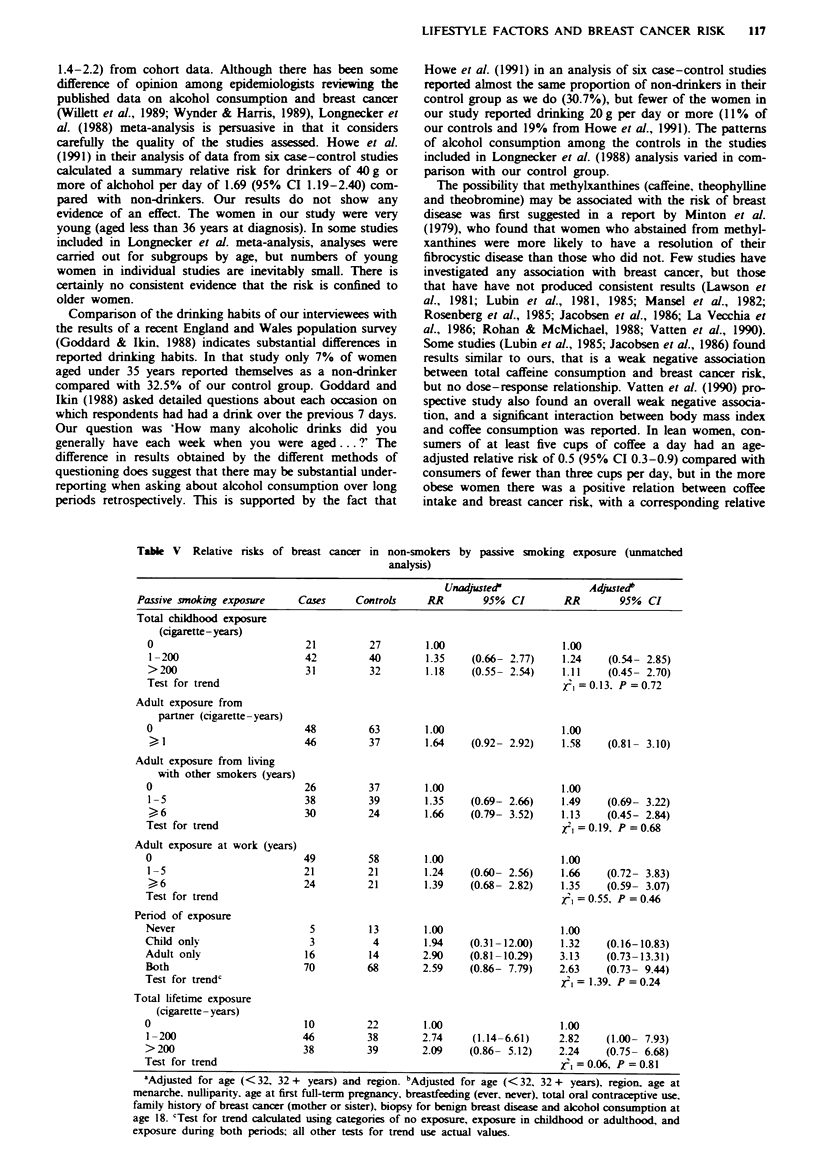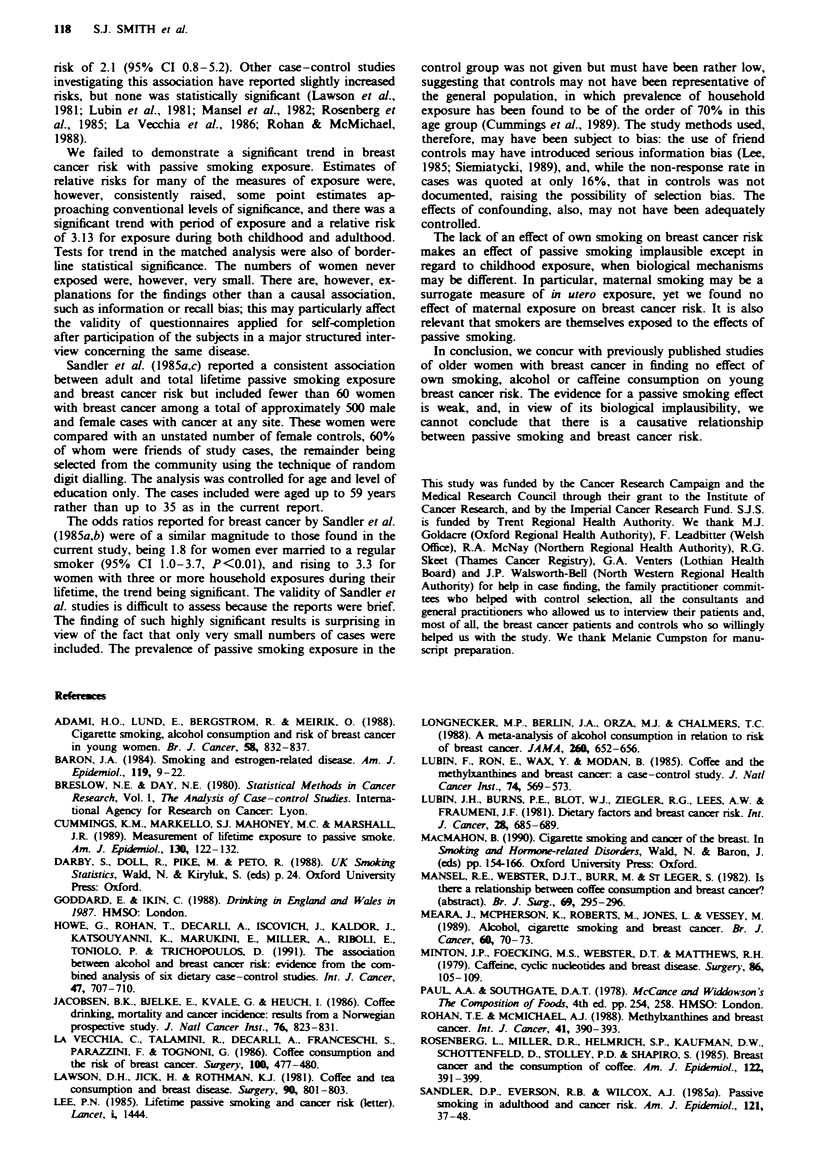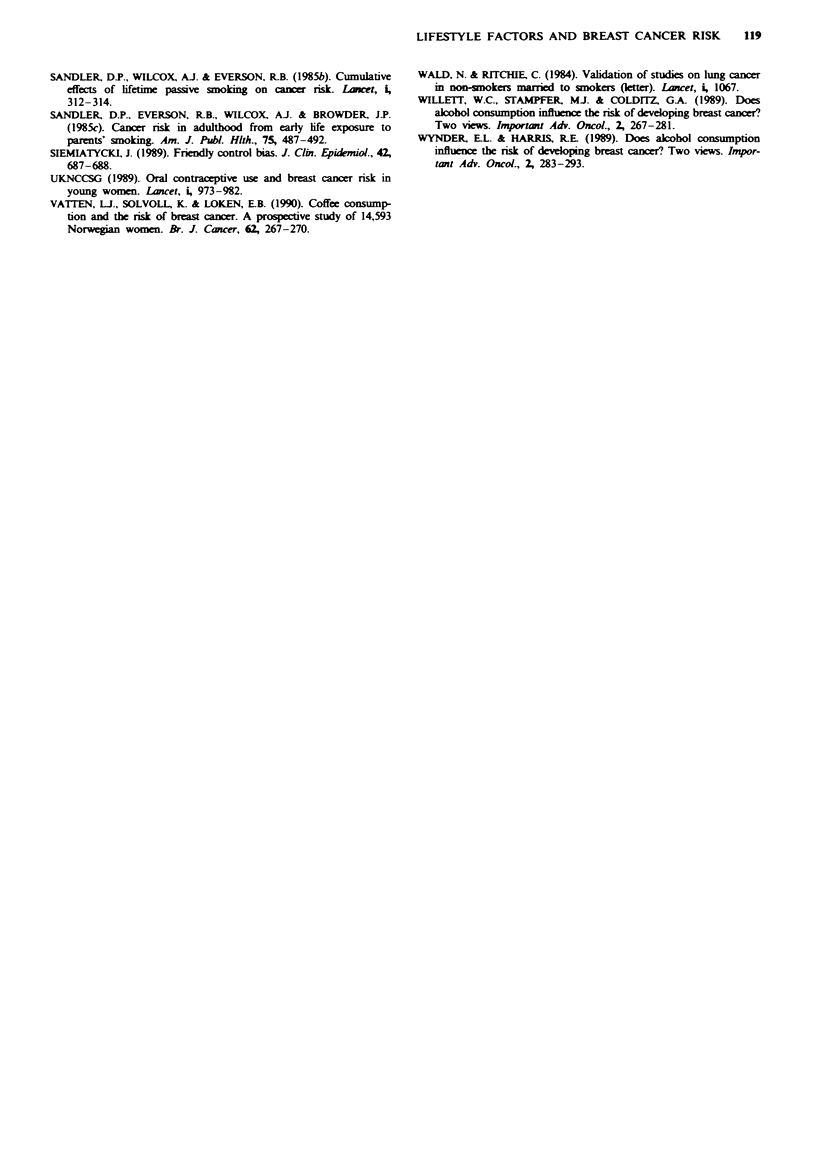# Alcohol, smoking, passive smoking and caffeine in relation to breast cancer risk in young women. UK National Case-Control Study Group.

**DOI:** 10.1038/bjc.1994.258

**Published:** 1994-07

**Authors:** S. J. Smith, J. M. Deacon, C. E. Chilvers

**Affiliations:** Department of Public Health Medicine, and Epidemiology, University of Nottingham Medical School, Queen's Medical Centre, UK.

## Abstract

The UK National Case-Control Study Group has examined the relationship between smoking (both own smoking and passive), alcohol consumption and caffeine consumption and the risk of breast cancer. A total of 755 women with breast cancer diagnosed before the age of 36, each with an age-matched general population control, were interviewed, and detailed information on reproductive, contraceptive and medical history, personal attributes and habits were obtained. Additional data on passive smoking were obtained from a subgroup of women. There was no evidence of a statistically significant difference in breast cancer risk between subjects who had ever smoked as much as one cigarette per day and those who had not [relative risk (RR) = 1.01, 95% confidence interval (CI) 0.81-1.26]. Most relative risks for passive smoking exceeded unity, but there was little evidence of significant trends with increasing exposure. The lack of effect of own smoking, and the fact that such smokers are also themselves exposed to the effects of passive smoking, makes any relationship between exposure to others' smoking and breast cancer risk implausible. Alcohol consumption during the year prior to diagnosis and at ages 18 and 25 was examined. Consumers of 0.1-4.9 and 5.0-14.9 g per day generally had non-significantly increased risks compared with never drinkers, but consumers of more than 15 g per day had reduced risks.


Br. J. Cancer (1994), 70, 112–119 © Macmillan Press Ltd., 1994

### Alcohol, smoking, passive smoking and caffeine in relation to breast cancer risk in young women

S.J. Smith[1], J.M. Deacon[2], C.E.D. Chilvers[1] and members of the UK National Case–Control Study Group*

[1]*Department of Public Health Medicine, and Epidemiology, University of Nottingham Medical School, Queen's Medical Centre, Nottingham, NG7 2UH, UK;* [2]*Section of Epidemiology, Institute of Cancer Research, Sutton, Surrey, SM2 5NG, UK.*

**Summary** The UK National Case–Control Study Group has examined the relationship between smoking (both own smoking and passive), alcohol consumption and caffeine consumption and the risk of breast cancer. A total of 755 women with breast cancer diagnosed before the age of 36, each with an age-matched general population control, were interviewed, and detailed information on reproductive, contraceptive and medical history, personal attributes and habits were obtained. Additional data on passive smoking were obtained from a subgroup of women. There was no evidence of a statistically significant difference in breast cancer risk between subjects who had ever smoked as much as one cigarette per day and those who had not [relative risk (RR) = 1.01, 95% confidence interval (CI) 0.81–1.26]. Most relative risks for passive smoking exceeded unity, but there was little evidence of significant trends with increasing exposure. The lack of effect of own smoking, and the fact that such smokers are also themselves exposed to the effects of passive smoking, makes any relationship between exposure to others' smoking and breast cancer risk implausible. Alcohol consumption during the year prior to diagnosis and at ages 18 and 25 was examined. Consumers of 0.1–4.9 and 5.0–14.9 g per day generally had non-significantly increased risks compared with never drinkers, but consumers of more than 15 g per day had reduced risks.

The UK National Case–Control Study Group (UKNCCSG) was set up primarily to investigate the relationship between oral contraceptive use and breast cancer risk in young women (UKNCCSG, 1989). Data were also collected on lifestyle factors such as smoking, alcohol consumption and caffeine consumption. We also investigated the relationship between breast cancer risk and passive smoking in response to findings reported by Sandler *et al.* (1985*a–c*), who found passive smoking to be a significant risk factor for breast cancer. For this part of the study an additional questionnaire on lifetime passive smoking exposure was sent to a subset of women in the main study.

### Materials and methods

### *Main study*

The study protocol and the statistical methods used have been described in detail elsewhere (UKNCCSG, 1989). Briefly, all women who were diagnosed as having breast cancer between 1982 and 1985 and who were resident in any of 11 health regions in the UK were included, provided that their breast cancer diagnosis was before their 36th birthday. For every case, one control was chosen, effectively at random, from the list of that case's general practitioner (GP). The control's date of birth was matched to within 6 months of the date of birth of the case, and the control had to have been registered with the GP before the date of diagnosis of the case. If a case could not be interviewed, no attempt was made to interview her matched control. If the chosen control could not be interviewed a second (or further) control was selected in the same manner as the first. For both cases and controls, the study was restricted to white women with no previous malignancy, severe mental handicap or psychiatric condition. The women were seen in their homes by trained interviewers between January 1984 and February 1988. Each case–control pair was interviewed by the same interviewer.

Every control was given a 'pseudodiagnosis' date, the date on which she was exactly the same age as her matching case was at diagnosis. The data analysed were mainly restricted to events before the diagnosis/pseudodiagnosis date, but some results are given for reference age (1 year before diagnosis/reference age). Pregnancy and contraceptive histories were elicited by constructing a calendar of events for each month from age 14 to diagnosis/pseudodiagnosis. After interview, data on obstetric and contraceptive history were abstracted from GP notes by trained interviewers, and contraceptive information was also sought from any family planning clinic that the women recalled attending. The data from all sources were used to construct a lifetime c[illegible]traceptive calendar.

Information on the smoking and drinking habits of subjects was also obtained at interview. Subjects were asked whether they had ever smoked as much as one cigarette a day for as long as 1 year, and, if so, the age at which they started smoking, the number of cigarettes smoked per day and the total number of years smoked. As a measure of lifetime exposure to cigarettes, a summary variable, cigarette–years, was calculated by multiplying the number of cigarettes smoked per day by the number of years smoked.

Total alcohol consumption was examined at three different times. The amount of alcohol consumed on average per week during the year prior to diagnosis, and at ages 25 and 18, was elicited. Subjects were asked how many alcoholic drinks, and of which type (beer, wine or spirits), they generally consumed per week. For each type of beverage, the alcoholic concentration (g $ml^{-1}$) as estimated by McCance and Widowson (1978) was multiplied by the quantity of beverage (ml) consumed, and the results for each type of drink were summed, to give the total amount of alcohol consumed in grams per day. Alcoholic content was calculated on the basis of

Correspondence: S.J. Smith.
*Principal investigators: Professor C.E.D. Chilvers, Professor K. McPherson, Professor J. Peto, Professor M.C. Pike and Professor M.P. Vessey.
Study Co-ordinators: Barbara Crossley, Carol Hermon and Cynthia Taylor.
Regional Collaborators: R.A. Cartwright (Leukaemia Research Fund Centre, University of Leeds); J.O.P. Chamberlain (South West Thames Regional Cancer Organisation, Sutton); P.C. Elwood [MRC Epidemiology Unit (South Wales)]; C.R. Gillis (West of Scotland Surveillance Unit, Greater Glasgow Health Boards); M.M. Roberts (Breast Screening Clinic, Edinburgh); M. Slattery (Wessex Regional Health Authority); A. Smith (Department of Epidemiology and Social Oncology, University of Manchester); J.H. Walker (Department of Family and Community Medicine, University of Newcastle upon Tyne).
Received 13 September 1993; and in revised form 26 January 1994.

100 ml of beer = 3.1 g of alcohol, 100 ml of wine = 9.4 g of alcohol and 100 ml of spirits = 31.7 g of alcohol.

Caffeine consumption at ages 16 and 25 was determined by asking subjects how many cups (or mugs) of tea and coffee (ground or instant) they consumed per day and how many cans of cola they consumed per week. Total caffeine consumption was estimated on the basis that tea contained 30 mg per cup, ground coffee 85 mg per cup, instant coffee 60 mg per cup and cola 45 mg per cup.

*Passive smoking study*

Supplementary information on passive smoking exposure was obtained from study participants who were resident in three of the participating health regions: South Thames, Oxford and the North-West. Data collection began in August 1985 and continued until the end of the main study in February 1988. Women eligible for the study were all those case-control pairs interviewed in the above regions after June 1984 who had consented to be recontacted, and whose interviewers were still working on the study. For women already interviewed, permission from their general practitioners was sought before they were recontacted to confirm that they were well enough to be included in the study.

Information on passive smoking exposure was obtained using a self-completion questionnaire, left with women after interview or mailed to those for whom interviews had already been completed. Questionnaires were returned by mail in reply-paid envelopes. Questions elicited information on passive smoking in childhood up to the age of 16 or of leaving school (whichever was earlier), and also on adult exposure from the partner (spouse, cohabitee) and from other sources, including work. When questionnaires were not returned within 3 weeks, a follow-up telephone call was made to the subject to encourage a positive response.

The information collected from those who had taken part in the passive smoking study was merged with selected data from the main oral contraceptive study to form a composite file for analysis. The three main exposures of interest were total childhood exposure, exposure from the partner during adult life and total cumulative lifetime exposure. Total childhood exposure was obtained by summing the number of cigarette-years of exposure, as defined by the number of cigarettes smoked per day in the home multiplied by the number of years of exposure, for every person in the household who smoked up until the time when the subject reached the age of 16 or left school. Adult exposure from the partner was measured in cigarette-years in the same way. Exposure from this source has been shown to be a good surrogate for total passive smoking exposure during adult life, since marriage to a smoker identifies a group of individuals who are most likely to be exposed from any source (Wald & Ritchie, 1984). Total lifetime exposure was obtained as the sum of the cigarette-years of exposure during childhood and the cigarette-years of exposure from the partner during adult life. A crude summary measure of period of lifetime passive smoking exposure (childhood/adulthood) was also calculated using the method reported by Sandler *et al.* (1985*b*): women were categorised according to the timing of their exposure, that is never exposed, exposed only during childhood, exposed only during adulthood or exposed during both periods of life. Other exposures examined to see if they were independently important were exposure from each parent (measured separately) during childhood, exposure from smokers other than the partner living in the home in adult life and exposure at work in adult life.

*Statistical methods*

The statistical analysis generally used multivariate logistic regression methods (Breslow & Day, 1980). Relative risks (RRs) were estimated by odds ratios with 95% confidence intervals (CIs). Analysis of the main study variables was matched; a case-control pair was excluded if information for either the case or the control was missing for the variable in question, and this explains the disparity between totals in the tables and the total number of responders.

Significance levels quoted are two-sided. Tests for trends across the own smoking and alcohol variables were calculated across categories of exposure. (The number of cigarettes smoked per day was consistently recorded in multiples of 5 or 10 according to the size of pack purchased, and trend tests for alcohol consumption using actual values were unduly influenced by a few control women with very high consumption levels.) Caffeine and passive smoking exposures that were measured as continuous variables used tests for trend fitted to the actual data values.

*Passive smoking study analysis*

For the passive smoking study a matched analysis and an unmatched analysis (controlling for age and region of residence in addition to the risk factors described below) was performed. The latter was carried out to increase the number of cases and controls available for the analysis since information was not always available for both members of a matched pair. The characteristics of the cases and controls in the passive smoking study were very similar to those in the main study. We previously reported statistically significant differences between cases and controls for a number of non-parity-related risk factors, namely age at menarche, family history of breast cancer and a history of biopsy for benign breast disease, and also for oral contraceptive use and breastfeeding (UKNCCSG, 1989). In the passive smoking study, significant differences between cases and controls were found in the crude relative risks for family history of breast cancer, parity and history of breastfeeding. Relative risks associated with oral contraceptive use were also very similar to the results of the main study, but did not reach conventional levels of significance because of the smaller numbers. All these factors, and the established parity-related risk factors for breast cancer, have been adjusted for in our analysis. Because of the possibly complex effects of confounding and interaction between active and passive smoking, the risks associated with passive smoking were also examined in an unmatched analysis of non-smokers alone.

### Results

*Response rates*

The response rates for the main study have been reported elsewhere (UKNCCSG, 1989). A total of 755 (71.9%) of the 1,049 eligible cases and 675 (89.4%) of the 755 first controls were interviewed; the remaining 80 controls were replaced by second (68) or subsequent (12) choices. For the passive smoking study, 327 (83.2%) of the 393 interviewed case-control pairs resident in the three study regions were eligible for recontact. Of these 260 pairs (79.5%) received passive smoking questionnaires. Reasons for exclusion were death of the case (13; 4.0%), case unwell (8; 2.4%), case or control moved from area (5; 1.5%), recontact refused by case or control (2; 0.6%) or change in study personnel (39; 11.9%). Of the cases 208/260 (80.0%) and of the controls 201/260 (77.3%) returned completed questionnaires either immediately or after a first reminder. Of these, information was available for 170 matched pairs (65.4%).

*Smoking*

When questioned about smoking habits 53.7% (403/751) of cases and 52.7% (396/751) of controls reported ever having smoked as much as one cigarette a day for as long as 1 year. Table I gives RRs by own smoking history. Adjustment for potential confounding factors listed in the footnote to Table I made little difference to the risk estimates, although all were slightly reduced. There was no evidence of a statistically significant difference in risk between subjects who had ever smoked as much as one cigarette a day and those who had

**Table I** Relative risks of breast cancer by own smoking history

| Exposure | Cases | Controls | Unadjusted RR | Unadjusted 95% CI | Adjusted[a] RR | Adjusted[a] 95% CI |
|---|---|---|---|---|---|---|
| Ever smoked | | | | | | |
| No | 348 | 355 | 1.00 | | 1.00 | |
| Yes | 403 | 396 | 1.04 | (0.85-1.28) | 1.01 | (0.81-1.26) |
| Age started smoking (years) | | | | | | |
| Never a smoker | 348 | 355 | 1.00 | | 1.00 | |
| 17+ | 196 | 206 | 0.97 | (0.75-1.24) | 0.93 | (0.71-1.22) |
| ⩽16 | 207 | 190 | 1.11 | (0.87-1.43) | 1.10 | (0.83-1.44) |
| Test for trend | | | | | $\chi^2_1 = 0.24$, $P = 0.63$ | |
| No. of cigarettes per day | | | | | | |
| Never a smoker | 348 | 355 | 1.00 | | 1.00 | |
| ⩽15 | 212 | 226 | 0.96 | (0.76-1.23) | 0.95 | (0.74-1.23) |
| 16+ | 191 | 170 | 1.16 | (0.89-1.50) | 1.10 | (0.83-1.45) |
| Test for trend | | | | | $\chi^2_1 = 0.26$, $P = 0.61$ | |
| Total years smoked | | | | | | |
| Never a smoker | 348 | 355 | 1.00 | | 1.00 | |
| 1-9 | 137 | 124 | 1.12 | (0.85-1.49) | 1.09 | (0.80-1.47) |
| 10+ | 266 | 272 | 1.00 | (0.79-1.25) | 0.97 | (0.76-1.24) |
| Test for trend | | | | | $\chi^2_1 = 0.04$, $P = 0.83$ | |
| Total amount smoked (cigarette - years) | | | | | | |
| Never a smoker | 348 | 355 | 1.00 | | 1.00 | |
| 1-200 | 236 | 239 | 1.01 | (0.80-1.27) | 1.00 | (0.78-1.29) |
| 200+ | 167 | 157 | 1.09 | (0.83-1.43) | 1.02 | (0.76-1.37) |
| Test for trend | | | | | $\chi^2_1 = 0.02$, $P = 0.90$ | |

[a]Adjusted for age at menarche, nulliparity, age at first full-term pregnancy, breastfeeding (ever, never), family history of breast cancer (mother or sister), total oral contraceptive use, biopsy for benign breast disease, total alcohol consumption at age 18.

not (RR = 1.01, 95% CI 0.81-1.26), or for any of the other smoking variables examined.

*Alcohol consumption*

Among cases and controls, 30.8% (232/753) and 32.5% (245/753) respectively reported having consumed no alcohol regularly during the year prior to diagnosis. The figures for non-drinkers among cases at ages 25 and 18 were 33.9% (245/722) and 35.3% (265/751) respectively, and for controls were 40.0% (289/722) and 34.6% (260/751) respectively.

The mean amount of alcohol consumed per day by drinkers in the control group was similar at each of the three ages (10.7 g day$^{-1}$ at reference age and at age 25, and 11.1 g day$^{-1}$ at age 18), although the range in quantity of alcohol consumed was greatest at age 18 (0-134.4 g day$^{-1}$ compared with 0-94.6 g day$^{-1}$ at age 25 and 0-89.6 g day$^{-1}$ at reference age). Coefficients of correlation indicated that drinking habits at reference age were much more similar to those at age 25 ($r = 0.52$) than to those at age 18 ($r = 0.21$).' The correlation coefficient between consumption at ages 25 and 18 was 0.29.

Table II gives RRs for total alcohol consumption at each age. Adjustment for potential confounders reduced risk estimates slightly. Heavy drinkers during the year prior to diagnosis appeared to have a reduced risk of developing breast cancer, consumers of 15 g or more per day having a RR of 0.66 (95% CI 0.45-0.96). This may have arisen because of a few particularly heavy drinkers in the control group [1.5% of controls drank 48 g day$^{-1}$ or more (an average of about four drinks per day) compared with 0.8% of cases]. The test for trend in quantity of alcohol consumed was not statistically significant ($\chi^2_1 = 1.93$, $P = 0.17$). The apparently reduced risk in consumers of 15 g or more per day was not statistically significant at ages 25 and 18. Beer, wine and spirit consumptions were considered separately at each age in order to determine whether there was a relationship between type of alcohol consumed and risk of breast cancer (data not shown). There was little evidence of any differential effect.

An unmatched analysis was also carried out by selecting women with consistent alcohol consumption at ages 18 and 25, and at ages 25 and reference age, in the latter case restricting the analysis to women diagnosed at age 30 or over (Table II). Relative risks were slightly higher in women with consistent consumption of 0.1-4.9 or 5.0-14.9 g day$^{-1}$ at ages 18 and 25 than when consumption was measured at a single time point, but there was still a low risk in the heaviest consumption group. None of the raised risk was statistically significant.

*Caffeine consumption*

Table III gives the RRs for total caffeine consumption at ages 25 and 16. Few subjects reported consuming no caffeine per day (at 25 years, seven cases and seven controls consumed no caffeine, and at 18 years, 26 cases and 22 controls consumed no caffeine) so a baseline category of consumption of 0-100 mg day$^{-1}$ was chosen to increase numbers in the reference group.

There was no evidence of a significant association between caffeine consumption and breast cancer risk, and no apparent trend in the amount of caffeine consumed at either age. At each level of caffeine consumption above the baseline level, however, risks of breast cancer were reduced, although not significantly so. Adjustment for confounding factors brought risk estimates closer to unity.

*Passive smoking*

The results of the ummatched analysis for the complete data set of 409 women are given in Table IV. Most relative risks for passive exposure to cigarette smoke were slightly in excess of unity. For total lifetime exposure, risks were statistically significantly raised, but there was no evidence of a trend with increasing exposure. The increased relative risks were due to a deficit of cases who had never been exposed (16 cases and 28 controls never exposed in childhood or from a partner) rather than an increasing risk with increasing exposures. There was no evidence of a significant trend for any of the individual exposure variables examined. The trend test for period of exposure (classified as never, childhood, adult or both childhood and adult exposure) was significant ($\chi^2_1 = 3.76$, $P = 0.05$), again because of a deficit of cases never exposed

(six cases and 14 controls never exposed at any time). The relative risk from exposure to maternal smoking was close to unity (RRs = 0.98 and 0.99 for 1–200 and more than 200 cigarette years of exposure respectively).

The matched analysis of 170 case–control pairs gave slightly lower RRs generally but marginally significant trends with increasing exposure in childhood ($\chi^2_1 = 3.55$, $P = 0.06$) and over the total lifetime ($\chi^2_1 = 3.51$, $P = 0.06$) were found. RRs for more than 400 cigarette–years of exposure in childhood and over the whole lifetime were 1.90 (95% CI 0.73–4.92) and 2.54 (95% CI 0.88–7.36) respectively.

Interactions between total childhood exposure and own smoking history and between total lifetime exposure and own smoking history were also investigated. There was no statistical evidence for heterogeneity between smokers and non-smokers for either of these two exposures (data not shown).

The results from the unmatched analysis in non-smokers,

**Table II** Relative risks of breast cancer by total alcohol consumption

| Exposure | Cases | Controls | Unadjusted RR | Unadjusted 95% CI | Adjusted[a] RR | Adjusted[a] 95% CI |
|---|---|---|---|---|---|---|
| Total alcohol consumption ($g\,day^{-1}$) at reference age | | | | | | |
| 0 | 232 | 245 | 1.00 | | 1.00 | |
| 0.1–4.9 | 210 | 188 | 1.18 | (0.90–1.55) | 1.15 | (0.86–1.53) |
| 5.0–14.9 | 224 | 206 | 1.14 | (0.88–1.49) | 1.08 | (0.81–1.43) |
| 15.0 + | 87 | 114 | 0.80 | (0.57–1.13) | 0.66 | (0.45–0.96) |
| Test for trend | | | | | $\chi^2_1 = 1.93$, $P = 0.17$ | |
| Total alcohol consumption ($g\,day^{-1}$) at age 25 years | | | | | | |
| 0 | 245 | 289 | 1.00 | | 1.00 | |
| 0.1–4.9 | 181 | 160 | 1.36 | (1.02–1.81) | 1.30 | (0.97–1.76) |
| 5.0–14.9 | 208 | 175 | 1.41 | (1.08–1.84) | 1.29 | (0.97–1.72) |
| 15.0 + | 88 | 98 | 1.09 | (0.78–1.52) | 0.96 | (0.67–1.40) |
| Test for trend | | | | | $\chi^2_1 = 0.31$, $P = 0.58$ | |
| Total alcohol consumption ($g\,day^{-1}$) at age 18 years | | | | | | |
| 0 | 265 | 260 | 1.00 | | 1.00 | |
| 0.1–4.9 | 168 | 167 | 0.98 | (0.74–1.30) | 0.95 | (0.71–1.28) |
| 5.0–14.9 | 218 | 211 | 1.02 | (0.78–1.31) | 0.99 | (0.76–1.31) |
| 15.0 + | 100 | 113 | 0.86 | (0.61–1.19) | 0.83 | (0.58–1.18) |
| Test for trend | | | | | $\chi^2_1 = 0.52$, $P = 0.47$ | |
| Total alcohol consumption at ages 18 and 25 | | | | | | |
| 0 | 136 | 162 | 1.00[b] | | 1.00[c] | |
| 0.1–4.9 | 53 | 46 | 1.46 | (0.91–2.34) | 1.28 | (0.77–2.12) |
| 5.0–14.9 | 91 | 75 | 1.50 | (1.01–2.23) | 1.31 | (0.86–2.00) |
| 15.0 + | 27 | 39 | 0.83 | (0.47–1.47) | 0.58 | (0.31–1.10) |
| Test for trend | | | | | $\chi^2_1 = 0.10$, $P = 0.76$ | |
| Total alcohol consumption at 25 and reference age – women diagnosed at ages 30–35 | | | | | | |
| 0 | 149 | 157 | 1.00[b] | | 1.00[c] | |
| 0.1–4.9 | 71 | 65 | 1.21 | (0.80–1.83) | 1.16 | (0.76–1.79) |
| 5.0–14.9 | 102 | 85 | 1.27 | (0.87–1.85) | 1.22 | (0.82–1.83) |
| 15.0 + | 40 | 38 | 1.14 | (0.68–1.91) | 0.83 | (0.47–1.46) |
| Test for trend | | | | | $\chi^2_1 = 0.02$, $P = 0.90$ | |

[a]Adjusted for age at menarche, nulliparity, age at first full-term pregnancy, breastfeeding (ever, never), family history of breast cancer (mother or sister), total oral contraceptive use, biopsy for benign breast disease, ever smoked (no, yes). [b]Unmatched analysis, adjusted for age (in single years) and region. [c]Unmatched analysis, adjusted for age (in single years), region and other factors as in footnote a.

**Table III** Relative risks of breast cancer by total caffeine consumption

| Exposure | Cases | Controls | Unadjusted RR | Unadjusted 95% CI | Adjusted[a] RR | Adjusted[a] 95% CI |
|---|---|---|---|---|---|---|
| Total caffeine consumption ($mg\,day^{-1}$) at age 25 | | | | | | |
| 0–100 | 35 | 26 | 1.00 | | 1.00 | |
| 101–200 | 107 | 122 | 0.66 | (0.38–1.17) | 0.67 | (0.37–1.21) |
| 201–300 | 180 | 178 | 0.76 | (0.43–1.33) | 0.82 | (0.46–1.49) |
| 301 + | 400 | 396 | 0.76 | (0.44–1.30) | 0.82 | (0.47–1.46) |
| Test for trend | | | | | $\chi^2_1 = 0.05$, $P = 0.82$ | |
| Total caffeine consumption ($mg\,day^{-1}$) at age 16 | | | | | | |
| 0–100 | 169 | 147 | 1.00 | | 1.00 | |
| 101–200 | 258 | 249 | 0.89 | (0.67–1.18) | 0.93 | (0.69–1.26) |
| 201–300 | 183 | 197 | 0.79 | (0.58–1.08) | 0.89 | (0.64–1.23) |
| 301 + | 140 | 157 | 0.76 | (0.55–1.06) | 0.83 | (0.59–1.17) |
| Test for trend | | | | | $\chi^2_1 = 2.71$, $P = 0.10$ | |

[a]Adjusted for age at menarche, nulliparity, age at first full-term pregnancy, breastfeeding (ever, never), family history of breast cancer (mother or sister), total oral contraceptive use, biopsy for benign breast disease, total alcohol consumption at age 18, and smoking (ever, never).

in which there were 94 cases and 99 controls, were very similar to those for the whole dataset (Table V). Relative risks were consistently slightly elevated, but confidence intervals were wide and included unity. There was no evidence of a significant dose response for any of the exposure variables.

### Discussion

In this study we have failed to demonstrate an association between own smoking and breast cancer in young women. Risk estimates were close to unity, and there was no evidence of any dose-response relationships. We were also unable to detect a relationship between alcohol consumption and breast cancer risk at any of the ages studied. Although risks were reduced at each level of caffeine consumption considered, these were not significantly different from unity. Our findings are unlikely to be affected by selection bias since our study was population based and our response rates were reasonably high.

The percentage of never smokers in our study (47%) was similar to the national figure (48%) given by Darby et al. (1988), for women smokers in 1985, although the percentage of current smokers in our study was lower (29% compared with 35%). MacMahon (1990) has reviewed the published data on cigarette smoking and breast cancer. Case-control studies excluding those hospital-based studies which included among their controls those with smoking-related disease have a summary odds ratio of 1.12 (95% CI 1.06-1.19). Our results are compatible with these. The cohort studies reviewed had a summary odds ratio of 1.14 (95% CI 1.02-1.27). MacMahon (1990) reported no evidence of interaction with menopausal status. The data that we present here are from a study of women diagnosed with breast cancer while very young (less than 36 at diagnosis), and few data are available for such young women. Adami et al. (1988), in their population-based case-control study of women diagnosed with breast cancer before age 45, reported results very similar to ours. Meara et al. (1989) hospital-based study included a subgroup of women aged 25-44, and likewise no evidence of an effect was found. Baron (1984) has suggested that smoking might produce an anti-oestrogenic effect which might protect against oestrogen-related disease, yet little evidence of such an effect has been found in epidemiological studies.

Longnecker et al. (1988) meta-analysis of studies of alcohol consumption and breast cancer risk concluded that there was a positive association, with strong evidence for a dose-response relationship in both case-control and cohort studies. For example, the relative risks associated with consumption of 24 g (about two drinks) per day were 1.4 (95% CI 1.0-1.8) from case-control studies and 1.7 (95% CI

**Table IV** Relative risks of breast cancer by passive smoking exposure (unmatched analysis)

| | | | Unadjusted[a] | | Adjusted[b] | |
|---|---|---|---|---|---|---|
| *Passive smoking exposure* | *Cases* | *Controls* | *RR* | *95% CI* | *RR* | *95% CI* |
| Total childhood exposure (cigarette-years) | | | | | | |
| 0 | 34 | 40 | 1.00 | | 1.00 | |
| 1-200 | 80 | 86 | 1.09 | (0.62- 1.91) | 1.22 | (0.66- 2.26) |
| 201-400 | 55 | 39 | 1.69 | (0.90- 3.19) | 2.09 | (1.05- 4.16) |
| >400 | 36 | 34 | 1.26 | (0.64- 2.48) | 1.51 | (0.72- 3.20) |
| Test for trend | | | | | $\chi^2_1 = 1.46$, $P = 0.23$ | |
| Adult exposure from partner (cigarette-years) | | | | | | |
| 0 | 87 | 96 | 1.00 | | 1.00 | |
| 1-200 | 107 | 88 | 1.38 | (0.92- 2.09) | 1.36 | (0.86- 2.13) |
| >200 | 13 | 17 | 0.84 | (0.38- 1.85) | 1.14 | (0.48- 2.70) |
| Test for trend | | | | | $\chi^2_1 = 0.55$, $P = 0.46$ | |
| Adult exposure from living with other smokers (years) | | | | | | |
| 0 | 39 | 51 | 1.00 | | 1.00 | |
| 1-5 | 100 | 89 | 1.55 | (0.91- 2.63) | 1.61 | (0.90- 2.86) |
| 6-10 | 55 | 50 | 1.51 | (0.84- 2.73) | 1.53 | (0.78- 2.96) |
| ⩾11 | 13 | 11 | 1.70 | (0.66- 4.35) | 1.36 | (0.47- 3.91) |
| Test for trend | | | | | $\chi^2_1 = 1.34$, $P = 0.25$ | |
| Adult exposure at work (years) | | | | | | |
| 0 | 105 | 106 | 1.00 | | 1.00 | |
| 1-5 | 45 | 39 | 1.19 | (0.71- 2.00) | 1.30 | (0.74- 2.29) |
| 6-10 | 28 | 36 | 0.78 | (0.44- 1.39) | 0.66 | (0.35- 1.25) |
| ⩾11 | 30 | 20 | 1.59 | (0.83- 3.05) | 1.32 | (0.65- 2.69) |
| Test for trend | | | | | $\chi^2_1 = 0.06$, $P = 0.81$ | |
| Period of exposure | | | | | | |
| Never | 6 | 14 | 1.00 | | 1.00 | |
| Child only | 5 | 5 | 2.17 | (0.43-10.88) | 1.98 | (0.35-11.36) |
| Adult only | 28 | 26 | 2.66 | (0.87- 8.15) | 2.65 | (0.80- 8.83) |
| Both | 165 | 154 | 2.62 | (0.96- 7.14) | 3.13 | (1.05- 9.38) |
| Test for trend[c] | | | | | $\chi^2_1 = 3.76$, $P = 0.05$ | |
| Total lifetime exposure (cigarette-years) | | | | | | |
| 0 | 16 | 28 | 1.00 | | 1.00 | |
| 1-200 | 76 | 74 | 1.91 | (0.94- 3.88) | 2.30 | (1.04- 5.09) |
| 201-400 | 62 | 47 | 2.45 | (1.17- 5.16) | 3.88 | (1.66- 9.08) |
| >400 | 50 | 50 | 1.85 | (0.87- 3.94) | 2.73 | (1.14- 6.56) |
| Test for trend | | | | | $\chi^2_1 = 1.85$, $P = 0.17$ | |

[a]Adjusted for age (in single years) and region. [b]Adjusted for age (in single years), region, age at menarche, nulliparity, age at first full-term pregnancy, breastfeeding (ever, never), total oral contraceptive use, family history of breast cancer (mother or sister), own smoking, biopsy for benign breast disease and alcohol consumption at age 18. [c]Test for trend calculated using categories of no exposure, exposure in childhood or adulthood, and exposure during both periods; all other tests for trend use actual values.

1.4–2.2) from cohort data. Although there has been some difference of opinion among epidemiologists reviewing the published data on alcohol consumption and breast cancer (Willett *et al.*, 1989; Wynder & Harris, 1989), Longnecker *et al.* (1988) meta-analysis is persuasive in that it considers carefully the quality of the studies assessed. Howe *et al.* (1991) in their analysis of data from six case–control studies calculated a summary relative risk for drinkers of 40 g or more of alchohol per day of 1.69 (95% CI 1.19–2.40) compared with non-drinkers. Our results do not show any evidence of an effect. The women in our study were very young (aged less than 36 years at diagnosis). In some studies included in Longnecker *et al.* meta-analysis, analyses were carried out for subgroups by age, but numbers of young women in individual studies are inevitably small. There is certainly no consistent evidence that the risk is confined to older women.

Comparison of the drinking habits of our interviewees with the results of a recent England and Wales population survey (Goddard & Ikin, 1988) indicates substantial differences in reported drinking habits. In that study only 7% of women aged under 35 years reported themselves as a non-drinker compared with 32.5% of our control group. Goddard and Ikin (1988) asked detailed questions about each occasion on which respondents had had a drink over the previous 7 days. Our question was 'How many alcoholic drinks did you generally have each week when you were aged...?' The difference in results obtained by the different methods of questioning does suggest that there may be substantial under-reporting when asking about alcohol consumption over long periods retrospectively. This is supported by the fact that Howe *et al.* (1991) in an analysis of six case–control studies reported almost the same proportion of non-drinkers in their control group as we do (30.7%), but fewer of the women in our study reported drinking 20 g per day or more (11% of our controls and 19% from Howe *et al.*, 1991). The patterns of alcohol consumption among the controls in the studies included in Longnecker *et al.* (1988) analysis varied in comparison with our control group.

The possibility that methylxanthines (caffeine, theophylline and theobromine) may be associated with the risk of breast disease was first suggested in a report by Minton *et al.* (1979), who found that women who abstained from methylxanthines were more likely to have a resolution of their fibrocystic disease than those who did not. Few studies have investigated any association with breast cancer, but those that have have not produced consistent results (Lawson *et al.*, 1981; Lubin *et al.*, 1981, 1985; Mansel *et al.*, 1982; Rosenberg *et al.*, 1985; Jacobsen *et al.*, 1986; La Vecchia *et al.*, 1986; Rohan & McMichael, 1988; Vatten *et al.*, 1990). Some studies (Lubin *et al.*, 1985; Jacobsen *et al.*, 1986) found results similar to ours, that is a weak negative association between total caffeine consumption and breast cancer risk, but no dose–response relationship. Vatten *et al.* (1990) prospective study also found an overall weak negative association, and a significant interaction between body mass index and coffee consumption was reported. In lean women, consumers of at least five cups of coffee a day had an age-adjusted relative risk of 0.5 (95% CI 0.3–0.9) compared with consumers of fewer than three cups per day, but in the more obese women there was a positive relation between coffee intake and breast cancer risk, with a corresponding relative

**Table V** Relative risks of breast cancer in non-smokers by passive smoking exposure (unmatched analysis)

| | | | *Unadjusted*[a] | | *Adjusted*[b] | |
|---|---|---|---|---|---|---|
| *Passive smoking exposure* | *Cases* | *Controls* | *RR* | *95% CI* | *RR* | *95% CI* |
| Total childhood exposure (cigarette-years) | | | | | | |
| 0 | 21 | 27 | 1.00 | | 1.00 | |
| 1–200 | 42 | 40 | 1.35 | (0.66– 2.77) | 1.24 | (0.54– 2.85) |
| >200 | 31 | 32 | 1.18 | (0.55– 2.54) | 1.11 | (0.45– 2.70) |
| Test for trend | | | | | $\chi^2_1 = 0.13$, $P = 0.72$ | |
| Adult exposure from partner (cigarette-years) | | | | | | |
| 0 | 48 | 63 | 1.00 | | 1.00 | |
| ⩾1 | 46 | 37 | 1.64 | (0.92– 2.92) | 1.58 | (0.81– 3.10) |
| Adult exposure from living with other smokers (years) | | | | | | |
| 0 | 26 | 37 | 1.00 | | 1.00 | |
| 1–5 | 38 | 39 | 1.35 | (0.69– 2.66) | 1.49 | (0.69– 3.22) |
| ⩾6 | 30 | 24 | 1.66 | (0.79– 3.52) | 1.13 | (0.45– 2.84) |
| Test for trend | | | | | $\chi^2_1 = 0.19$, $P = 0.68$ | |
| Adult exposure at work (years) | | | | | | |
| 0 | 49 | 58 | 1.00 | | 1.00 | |
| 1–5 | 21 | 21 | 1.24 | (0.60– 2.56) | 1.66 | (0.72– 3.83) |
| ⩾6 | 24 | 21 | 1.39 | (0.68– 2.82) | 1.35 | (0.59– 3.07) |
| Test for trend | | | | | $\chi^2_1 = 0.55$, $P = 0.46$ | |
| Period of exposure | | | | | | |
| Never | 5 | 13 | 1.00 | | 1.00 | |
| Child only | 3 | 4 | 1.94 | (0.31–12.00) | 1.32 | (0.16–10.83) |
| Adult only | 16 | 14 | 2.90 | (0.81–10.29) | 3.13 | (0.73–13.31) |
| Both | 70 | 68 | 2.59 | (0.86– 7.79) | 2.63 | (0.73– 9.44) |
| Test for trend[c] | | | | | $\chi^2_1 = 1.39$, $P = 0.24$ | |
| Total lifetime exposure (cigarette-years) | | | | | | |
| 0 | 10 | 22 | 1.00 | | 1.00 | |
| 1–200 | 46 | 38 | 2.74 | (1.14–6.61) | 2.82 | (1.00– 7.93) |
| >200 | 38 | 39 | 2.09 | (0.86– 5.12) | 2.24 | (0.75– 6.68) |
| Test for trend | | | | | $\chi^2_1 = 0.06$, $P = 0.81$ | |

[a]Adjusted for age (<32, 32+ years) and region. [b]Adjusted for age (<32, 32+ years), region, age at menarche, nulliparity, age at first full-term pregnancy, breastfeeding (ever, never), total oral contraceptive use, family history of breast cancer (mother or sister), biopsy for benign breast disease and alcohol consumption at age 18. [c]Test for trend calculated using categories of no exposure, exposure in childhood or adulthood, and exposure during both periods; all other tests for trend use actual values.

risk of 2.1 (95% CI 0.8–5.2). Other case–control studies investigating this association have reported slightly increased risks, but none was statistically significant (Lawson *et al.*, 1981; Lubin *et al.*, 1981; Mansel *et al.*, 1982; Rosenberg *et al.*, 1985; La Vecchia *et al.*, 1986; Rohan & McMichael, 1988).

We failed to demonstrate a significant trend in breast cancer risk with passive smoking exposure. Estimates of relative risks for many of the measures of exposure were, however, consistently raised, some point estimates approaching conventional levels of significance, and there was a significant trend with period of exposure and a relative risk of 3.13 for exposure during both childhood and adulthood. Tests for trend in the matched analysis were also of borderline statistical significance. The numbers of women never exposed were, however, very small. There are, however, explanations for the findings other than a causal association, such as information or recall bias; this may particularly affect the validity of questionnaires applied for self-completion after participation of the subjects in a major structured interview concerning the same disease.

Sandler *et al.* (1985*a,c*) reported a consistent association between adult and total lifetime passive smoking exposure and breast cancer risk but included fewer than 60 women with breast cancer among a total of approximately 500 male and female cases with cancer at any site. These women were compared with an unstated number of female controls, 60% of whom were friends of study cases, the remainder being selected from the community using the technique of random digit dialling. The analysis was controlled for age and level of education only. The cases included were aged up to 59 years rather than up to 35 as in the current report.

The odds ratios reported for breast cancer by Sandler *et al.* (1985*a,b*) were of a similar magnitude to those found in the current study, being 1.8 for women ever married to a regular smoker (95% CI 1.0–3.7, $P<0.01$), and rising to 3.3 for women with three or more household exposures during their lifetime, the trend being significant. The validity of Sandler *et al.* studies is difficult to assess because the reports were brief. The finding of such highly significant results is surprising in view of the fact that only very small numbers of cases were included. The prevalence of passive smoking exposure in the control group was not given but must have been rather low, suggesting that controls may not have been representative of the general population, in which prevalence of household exposure has been found to be of the order of 70% in this age group (Cummings *et al.*, 1989). The study methods used, therefore, may have been subject to bias: the use of friend controls may have introduced serious information bias (Lee, 1985; Siemiatycki, 1989), and, while the non-response rate in cases was quoted at only 16%, that in controls was not documented, raising the possibility of selection bias. The effects of confounding, also, may not have been adequately controlled.

The lack of an effect of own smoking on breast cancer risk makes an effect of passive smoking implausible except in regard to childhood exposure, when biological mechanisms may be different. In particular, maternal smoking may be a surrogate measure of *in utero* exposure, yet we found no effect of maternal exposure on breast cancer risk. It is also relevant that smokers are themselves exposed to the effects of passive smoking.

In conclusion, we concur with previously published studies of older women with breast cancer in finding no effect of own smoking, alcohol or caffeine consumption on young breast cancer risk. The evidence for a passive smoking effect is weak, and, in view of its biological implausibility, we cannot conclude that there is a causative relationship between passive smoking and breast cancer risk.

This study was funded by the Cancer Research Campaign and the Medical Research Council through their grant to the Institute of Cancer Research, and by the Imperial Cancer Research Fund. S.J.S. is funded by Trent Regional Health Authority. We thank M.J. Goldacre (Oxford Regional Health Authority), F. Leadbitter (Welsh Office), R.A. McNay (Northern Regional Health Authority), R.G. Skeet (Thames Cancer Registry), G.A. Venters (Lothian Health Board) and J.P. Walsworth-Bell (North Western Regional Health Authority) for help in case finding, the family practitioner committees who helped with control selection, all the consultants and general practitioners who allowed us to interview their patients and, most of all, the breast cancer patients and controls who so willingly helped us with the study. We thank Melanie Cumpston for manuscript preparation.

### References

ADAMI, H.O., LUND, E., BERGSTROM, R. & MEIRIK, O. (1988). Cigarette smoking, alcohol consumption and risk of breast cancer in young women. *Br. J. Cancer*, **58**, 832–837.

BARON, J.A. (1984). Smoking and estrogen-related disease. *Am. J. Epidemiol.*, **119**, 9–22.

BRESLOW, N.E. & DAY, N.E. (1980). *Statistical Methods in Cancer Research*, Vol. 1, *The Analysis of Case–control Studies*. International Agency for Research on Cancer: Lyon.

CUMMINGS, K.M., MARKELLO, S.J. MAHONEY, M.C. & MARSHALL, J.R. (1989). Measurement of lifetime exposure to passive smoke. *Am. J. Epidemiol.*, **130**, 122–132.

DARBY, S., DOLL, R., PIKE, M. & PETO, R. (1988). *UK Smoking Statistics*, Wald, N. & Kiryluk, S. (eds) p. 24. Oxford University Press: Oxford.

GODDARD, E. & IKIN, C. (1988). *Drinking in England and Wales in 1987*. HMSO: London.

HOWE, G., ROHAN, T., DECARLI, A., ISCOVICH, J., KALDOR, J., KATSOUYANNI, K., MARUKINI, E., MILLER, A., RIBOLI, E., TONIOLO, P. & TRICHOPOULOS, D. (1991). The association between alcohol and breast cancer risk: evidence from the combined analysis of six dietary case–control studies. *Int. J. Cancer*, **47**, 707–710.

JACOBSEN, B.K., BJELKE, E., KVALE, G. & HEUCH, I. (1986). Coffee drinking, mortality and cancer incidence: results from a Norwegian prospective study. *J. Natl Cancer Inst.*, **76**, 823–831.

LA VECCHIA, C., TALAMINI, R., DECARLI, A., FRANCESCHI, S., PARAZZINI, F. & TOGNONI, G. (1986). Coffee consumption and the risk of breast cancer. *Surgery*, **100**, 477–480.

LAWSON, D.H., JICK, H. & ROTHMAN, K.J. (1981). Coffee and tea consumption and breast disease. *Surgery*, **90**, 801–803.

LEE, P.N. (1985). Lifetime passive smoking and cancer risk (letter). *Lancet*, **i**, 1444.

LONGNECKER, M.P., BERLIN, J.A., ORZA, M.J. & CHALMERS, T.C. (1988). A meta-analysis of alcohol consumption in relation to risk of breast cancer. *JAMA*, **260**, 652–656.

LUBIN, F., RON, E., WAX, Y. & MODAN, B. (1985). Coffee and the methylxanthines and breast cancer: a case–control study. *J. Natl Cancer Inst.*, **74**, 569–573.

LUBIN, J.H., BURNS, P.E., BLOT, W.J., ZIEGLER, R.G., LEES, A.W. & FRAUMENI, J.F. (1981). Dietary factors and breast cancer risk. *Int. J. Cancer*, **28**, 685–689.

MACMAHON, B. (1990). Cigarette smoking and cancer of the breast. In *Smoking and Hormone-related Disorders*, Wald, N. & Baron, J. (eds) pp. 154-166. Oxford University Press: Oxford.

MANSEL, R.E., WEBSTER, D.J.T., BURR, M. & ST LEGER, S. (1982). Is there a relationship between coffee consumption and breast cancer? (abstract). *Br. J. Surg.*, **69**, 295–296.

MEARA, J., MCPHERSON, K., ROBERTS, M., JONES, L. & VESSEY, M. (1989). Alcohol, cigarette smoking and breast cancer. *Br. J. Cancer*, **60**, 70–73.

MINTON, J.P., FOECKING, M.S., WEBSTER, D.T. & MATTHEWS, R.H. (1979). Caffeine, cyclic nucleotides and breast disease. *Surgery*, **86**, 105–109.

PAUL, A.A. & SOUTHGATE, D.A.T. (1978). *McCance and Widdowson's The Composition of Foods*, 4th ed. pp. 254, 258. HMSO: London.

ROHAN, T.E. & MCMICHAEL, A.J. (1988). Methylxanthines and breast cancer. *Int. J. Cancer*, **41**, 390–393.

ROSENBERG, L., MILLER, D.R., HELMRICH, S.P., KAUFMAN, D.W., SCHOTTENFELD, D., STOLLEY, P.D. & SHAPIRO, S. (1985). Breast cancer and the consumption of coffee. *Am. J. Epidemiol.*, **122**, 391–399.

SANDLER, D.P., EVERSON, R.B. & WILCOX, A.J. (1985*a*). Passive smoking in adulthood and cancer risk. *Am. J. Epidemiol.*, **121**, 37–48.

SANDLER, D.P., WILCOX, A.J. & EVERSON, R.B. (1985*b*). Cumulative effects of lifetime passive smoking on cancer risk. *Lancet*, **i**, 312–314.

SANDLER, D.P., EVERSON, R.B., WILCOX, A.J. & BROWDER, J.P. (1985*c*). Cancer risk in adulthood from early life exposure to parents' smoking. *Am. J. Publ. Hlth.*, **75**, 487–492.

SIEMIATYCKI, J. (1989). Friendly control bias. *J. Clin. Epidemiol.*, **42**, 687–688.

UKNCCSG (1989). Oral contraceptive use and breast cancer risk in young women. *Lancet*, **i**, 973–982.

VATTEN, L.J., SOLVOLL, K. & LOKEN, E.B. (1990). Coffee consumption and the risk of breast cancer. A prospective study of 14,593 Norwegian women. *Br. J. Cancer*, **62**, 267–270.

WALD, N. & RITCHIE, C. (1984). Validation of studies on lung cancer in non-smokers married to smokers (letter). *Lancet*, **i**, 1067.

WILLETT, W.C., STAMPFER, M.J. & COLDITZ, G.A. (1989). Does alcohol consumption influence the risk of developing breast cancer? Two views. *Important Adv. Oncol.*, **2**, 267–281.

WYNDER, E.L. & HARRIS, R.E. (1989). Does alcohol consumption influence the risk of developing breast cancer? Two views. *Important Adv. Oncol.*, **2**, 283–293.